# A novel computational analysis integrating social determinants information from EHR and literature with Alzheimer’s disease biological knowledge through large language models and knowledge graphs

**DOI:** 10.1093/geroni/igaf102

**Published:** 2025-09-23

**Authors:** Tianqi Shang, Shu Yang, Tianhua Zhai, Weiqing He, Elizabeth Mamourian, Jiayu Zhang, Bojian Hou, Joseph Lee, Duy Duong-Tran, Jason H Moore, Marylyn D Ritchie, Li Shen

**Affiliations:** Department of Biostatistics, Epidemiology and Informatics, University of Pennsylvania, Philadelphia, Pennsylvania, United States; Department of Biostatistics, Epidemiology and Informatics, University of Pennsylvania, Philadelphia, Pennsylvania, United States; Department of Biostatistics, Epidemiology and Informatics, University of Pennsylvania, Philadelphia, Pennsylvania, United States; Department of Mathematics, University of Pennsylvania, Philadelphia, Pennsylvania, United States; Department of Biostatistics, Epidemiology and Informatics, University of Pennsylvania, Philadelphia, Pennsylvania, United States; Department of the School of Engineering and Applied Science, University of Pennsylvania, Philadelphia, Pennsylvania, United States; Department of Biostatistics, Epidemiology and Informatics, University of Pennsylvania, Philadelphia, Pennsylvania, United States; Department of the School of Engineering and Applied Science, University of Pennsylvania, Philadelphia, Pennsylvania, United States; Department of Mathematics, United States Naval Academy, Annapolis, Maryland, United States; Department of Computational Biomedicine Cedars Sinai Medical Center, West Hollywood, California, United States; Department of Biostatistics, Epidemiology and Informatics, University of Pennsylvania, Philadelphia, Pennsylvania, United States; Department of Biostatistics, Epidemiology and Informatics, University of Pennsylvania, Philadelphia, Pennsylvania, United States

**Keywords:** Dementia, SDoH, Natural language processing, Machine learning

## Abstract

**Background and Objectives:**

Alzheimer’s disease (AD) and AD-related dementias (ADRD) are expected to affect over 100 million people by 2050, placing a significant strain on public health systems. Social determinants of health (SDoH), which include factors such as socioeconomic conditions and environment, play a crucial role in AD risk. Despite growing evidence, the understanding of SDoH’s impact on AD remains limited.

**Research Design and Methods:**

This study leverages large language models and knowledge graphs (KGs) to extract AD-related SDoH knowledge from literature and electronic health records (EHR). We integrate this knowledge into biological research on AD through KG construction and graph deep learning, performing KG-link predictions validated by multimodal biological data from single-cell RNA-seq and proteomics.

**Results:**

We generated an SDoH knowledge graph with around 92k triplets, integrating literature and EHR data. In various link prediction experiments, we observed higher accuracy when integrating SDoH into knowledge graphs. Additionally, exploratory predictions uncovered potential SDoH-gene interactions, many of which were validated through differential expression analysis using proteomics and RNA-seq data.

**Discussion and Implications:**

This novel KG-based analysis enhances link prediction in AD-related biomedical networks by integrating SDoH and biological knowledge. Our findings highlight the potential interaction between social determinants and biological factors in AD, offering insights into more personalized and socially aware healthcare interventions.

Translational Significance This study explored how social determinants influence the pathogenesis of Alzheimer’s disease (AD) by mining extensive electronic health records and literature data. Key outcomes include an augmented knowledge graph (KG) that integrates AD-related social determinants of health (SDoH) and biological knowledge together utilizing large language models and machine learning-based knowledge discovery on the KG to identify AD-related biological relationships and novel SDoH-gene interactions validated through proteomics and RNA-seq data. Our findings highlight potential interventions on social factors in AD etiology, emphasizing the importance of enhancing health equity and informing prevention strategies for AD progression.

## Background and objectives

Alzheimer’s disease (AD) is a complex neurodegenerative disease that is the single most common form of dementia worldwide and the sixth leading cause of death in the United States.^1^^,^^2^ The underlying etiology of AD remains unclear today, largely due to its heterogeneity, as multiple medical and nonmedical factors affect individuals differently.^3–5^ Social determinants of health (SDoH)—nonmedical factors defined by the World Health Organization as the “conditions in which people are born, grow, work, live, and age, and the wider set of forces and systems shaping the conditions of daily life”^6^—have been shown to explain much of the heterogeneity in cognitive, functional, biomarker, and interventional outcomes in AD and related dementias.^7^^,^^8^ It is estimated that SDoH broadly accounts for 80%-90% of modifiable health factors.^9^ For AD, limited pharmacological treatments currently available have prompted researchers to focus on identifying modifiable social determinant risk factors to prevent or delay disease progression.^10^^,^^11^ Previous studies reported that factors, such as low education level,^12^ food insecurity,^13^ low socioeconomic status,^14^ loneliness, and low social engagements,^15^ are associated with increased risk and accelerated cognitive decline in AD. However, little is known about the mechanisms linking SDoH to the biological processes of disease development. A major challenge here is the difficulty in collecting relevant information that lacks standardization and is dispersed among electronic health records (EHR), claims data, scientific literature, and other sources, which makes manual collection unrealistic and requires advanced, automated solutions.^16^^,^^17^

Computational approaches based on natural language processing (NLP) have been developed to automatically and efficiently extract SDoH information in health research.^18^^,^^19^ The vast majority of existing works focus on identifying SDoH from the rich source of EHRs, such as physician and nurse notes. They employ various methods ranging from simple rule-based searches^20^ to deep neural networks^21^ and recent large language models (LLMs).^22^ These methods have shown remarkable potential for mining SDoH from EHR by NLP, including in the context of AD.^20^ However, although EHRs contain rich SDoH information, they are predominantly unstructured narratives or free texts that only embed social determinants in a noisy way and are not standardized traditionally.^18^^,^^19^ In contrast, peer-reviewed scientific articles usually contain rigorous, high-quality SDoH information and their relations with other health concepts, though such information is not as abundant as in EHRs. A few recent studies sought to use literature to study SDoH and health outcomes,^17^^,^^23^ including AD.^24^ Among them, one notable study by Park et al.^17^ focused on two specific SDoH, housing and unemployment, and merged individual knowledge graphs (KGs) constructed from literature and EHR to explore the associations between the two determinants and health outcomes like diabetes and hypertension.

In addition, knowledge graphs have attracted growing interest in Alzheimer’s research. Several studies^25–27^ recently used literature mining approaches to extract knowledge triplets from scientific papers. For instance, Pu et al. used conventional NLP methods to derive triplets from an expert collection of AD literature and evaluated the KG on link prediction tasks using different graph embedding-based learning methods.^25^ Using the same AD literature corpus, Li et al. leveraged LLMs to construct KGs and retrieve knowledge triplets from them through a dynamic co-augmentation framework.^26^ Some other works^28–30^ draw from a variety of heterogeneous databases and other sources to construct KGs, instead of just literature alone. As a representative example, Alzheimer’s Knowledge Base (AlzKB),^30^ recently published in 2024, is by far the largest KG dedicated to AD and has adopted this strategy to provide a comprehensive knowledge representation of AD etiology and candidate therapeutics.

Here, we present a systematic analysis of AD-related SDoH knowledge extracted from EHR and literature using our novel framework. This approach employs pre-trained LLMs to facilitate triplet extraction and KG construction, and it further integrates the resultant SDoH KG with a widely-used biological KG^31^ to explore connections with disease biological processes. Our main contributions include (a) extracting and integrating SDoH knowledge from EHR and literature via an automated pipeline using pre-trained LLMs and advanced NLP techniques; (b) integrating extracted AD-related SDoH knowledge triplets with entities in PrimeKG to create a heterogeneous AD KG augmented with social determinants information; (c) utilizing our novel KG for knowledge discovery through link predictions using graph convolutional networks (GCNs); and (d) validating our prediction results with multimodal single-cell RNA-seq and proteomics data. As noted earlier, as far as we know, there is only one previous study^17^ that also combines KGs from literature and EHRs together for SDoH research. Our work is fundamentally different in several aspects by integrating the SDoH KG further with biological knowledge in PrimeKG, covering a comprehensive list of SDoH factors, validating through biological data, utilizing LLMs for KG construction, and focusing on AD. To the best of our knowledge, our systematic analysis is the first study combining SDoH knowledge from EHR and literature together with biological knowledge from existing KG for AD research, and it may open new opportunities for deciphering AD etiology.

The rest of the paper is organized as follows: First, we describe the data used in this study. Next, we detail the main steps of our LLM-driven text mining method, which (a) extracts knowledge triplets from literature and EHR, respectively, to construct preliminary SDoH KGs, (b) integrates SDoH knowledge with biological knowledge from the existing PrimeKG, and (c) performs downstream link predictions for knowledge discovery. We then report the key experimental results of our KG-based link prediction analysis for uncovering SDoH-gene relationships in AD. We also present the subsequent validations with real-world biological data from single-cell gene expression and recent proteomics experiments. We conclude by discussing the interpretation and implications of our findings, as well as potential future extensions of the analytic framework.

## Research design and methods

### Dataset

#### EHR and literature corpus

We integrated data from two primary sources to construct the knowledge graph: the MIMIC-III database^32^ and the PubMed repository. We gathered 12,733 articles published within the last 5 years, focusing on both AD and SDoH from PubMed and extracted data for 686 patients diagnosed with AD or related conditions, identified by ICD-9 codes: 3310, 2900, 2901, 2902, 2903, 2904, 3311, 3312, 33182, 2941, and 4380, from MIMIC-III. For the MIMIC-III patients, we also included ADRD subjects since the AD-only cases are very limited in MIMIC-III, and ADRD share many key cognitive and pathological features with AD. For each patient, the following tables were retrieved: ADMISSIONS, DIAGNOSES_ICD, NOTEEVENTS, PATIENTS, and PRESCRIPTIONS. Detailed descriptions of these tables are available at MIMIC-III Documentation (https://mimic.mit.edu/docs/iii/tables/).

#### Proteomics and RNA-seq data

For the final part of our study, that is, to validate the findings of potentially SDoH-associated biological entities (details will be described later), we examined proteomics and single-cell RNA-seq data from several related studies for evidence. Proteomics data from the Accelerating Medicines Partnership for Alzheimer’s Disease (AMP-AD) Diverse Cohorts^33^ and the Religious Orders Study and Memory and Aging Project (ROSMAP) Round 1^34^ were downloaded (AD Knowledge Portal^35^ syn51757664, syn21261728) and analyzed to identify differentially abundant proteins in AD. The proteomics data from both sources come from post-mortem samples of the dorsolateral prefrontal cortex. We included all available subjects in the datasets, with a total of 894 participants from AMP-AD and 192 from ROSMAP (both with AD cases and controls). For the AMP-AD dataset, all AD donors had a definite diagnosis according to the NINCDS-ADRDA criteria and had Braak NFT stage of IV or greater. Controls donors had Braak NFT stage of III or less, CERAD neuritic and cortical plaque densities of 0 (none) or 1 (sparse), and lacked any of the following pathologic diagnoses: AD, Parkinson’s disease, Dementia with Lewy bodies (DLB), Vascular Dementia (VaD), Progressive supranuclear palsy (PSP), motor neuron disease, Corticobasal degeneration (CBD), Pick’s disease, Huntington’s disease, Frontotemporal lobar degeneration (FTLD), hippocampal sclerosis (HipScl), or dementia lacking distinctive histology. For the ROSMAP dataset, a subject’s AD status was determined by a computer algorithm based on cognitive test performance with a series of discrete clinical judgments made in series by a neuropsychologist and a clinician. More details can be found on the AD Knowledge Portal https://www.synapse.org/Synapse:syn59611693 and https://www.synapse.org/Synapse:syn21261728. The demographic details of the subjects can be found in Supplementary Table 2.

Moreover, we obtained processed single-cell RNA sequencing data from the Seattle Alzheimer’s Disease Brain Cell Atlas (SEA-AD). The dataset includes 84 high-quality subjects: 42 AD patients and 42 control subjects with no AD or low to intermediate dementia. Participants ranged in age from 65 to 102 years, with a mean age of 88. The study collected 1,395,601 cells from the dorsolateral prefrontal cortex of the brain, the same as the proteomics data.

### Overview of the method framework

**Figure 1.**
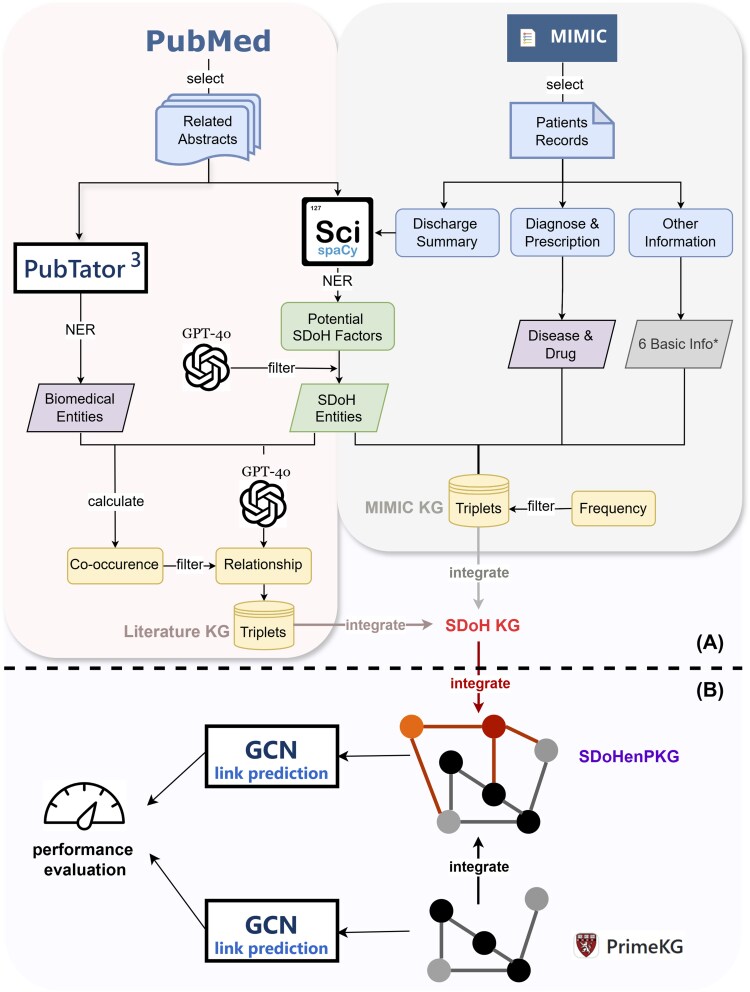
The schematic overview of our framework. KG = knowledge graph; AD = Alzheimer’s disease; SDoH = social determinants of health. Different colors are used here to indicate different KGs. (A) Workflow for generating an AD-related SDoH knowledge graph (SDoH KG, red color), combining triplets extracted from PubMed (Literature KG, pink color) and MIMIC-III datasets (MIMIC KG, silver color). The six basic information include age, ethnicity, religion, marital status, gender, and insurance. (B) Workflow for integrating SDoH KG, and PrimeKG (black/gray color) to construct SDoHenPKG (purple-colored fonts with red and black/gray colored KG), followed by the comparison of the SDoHenPKG and PrimeKG for link prediction tasks based on graph convolutional networks.

Figure 1 shows the schematic overview of the proposed framework: Figure 1A illustrates the workflow of how we generated an AD-related SDoH knowledge graph from the PubMed and MIMIC-III datasets; Figure 1B illustrates the integration of PrimeKG to construct SDoHenPKG and the performance evaluation via KG link predictions.

Here, we provide a brief introduction to the steps in the framework, and we will detail each step in the following sections. First, we collected relevant articles from PubMed and relevant patient records from EHR. From them, we performed named entity recognition (NER) to identify biomedical and SDoH entities using OpenAI’s GPT-4o and conventional NLP methods, and we then extracted potential relations among these recognized entities with GPT’s language capabilities and filtered for co-occurrences to ensure accuracy. This text mining step resulted in two different sets of SDoH triplets (i.e., two preliminary KGs, Literature KG and MIMIC KG, as in Figure 1A) with diverse knowledge from rigorous scientific research to real-world health records. They were complementary to each other by nature and together formed our SDoH KG. To investigate the SDoH AD risk and underlying etiology, we further integrated the SDoH KG with the widely used PrimeKG,^31^ a biological KG for drug repurposing that does not have any SDoH knowledge. As shown in Figure 1B, we merged the highly heterogeneous knowledge from PrimeKG and SDoH KG to provide a rich data resource, that is, the final knowledge graph SDoHenPKG, for AD-SDoH investigation. We assessed the impact of adding SDoH knowledge to PrimeKG on knowledge discovery in AD by performing graph embedding-based link predictions using graph convolutional networks. We compared the link prediction results of PrimeKG versus SDoHenPKG, demonstrating the utility of our augmented knowledge graph in identifying novel connections and offering new insights for research and intervention in AD.

The source codes and data (excluding non-public MIMIC data) for the framework are made publicly available at GitHub: https://github.com/PennShenLab/AlzSDOH_EHRlit_KGllm.

### SDoH knowledge graph construction

In this section, we will describe the detailed steps to construct the SDoH knowledge graphs from scientific literature and EHR data, as shown in Figure 1A.

#### SDoH entity extraction

Given the variability and irregularity of how SDoH are expressed across texts, training a universal model for extraction is challenging. While large language models excel in text processing, they pose privacy risks when handling patient-level EHR data. To address this issue, we used scispaCy,^36^ an NLP toolkit designed for biomedical text processing, to perform NER locally on the cleaned text from literature abstracts and patient discharge summaries. The text cleaning process involved standard preprocessing steps, such as removing punctuation, special characters, and extra whitespace, as well as converting all text to lowercase. These steps ensure that the text is in a consistent format, making it suitable for accurate NER and further analysis. The initial NER output generated a list of potential SDoH entities, which may still include irrelevant terms or incomplete phrases requiring further refinement. However, with all extracted entities aggregated, we could safely process them using LLMs without privacy concerns. Furthermore, in the case of the MIMIC-III discharge summaries, all extracted entities were aggregated, and subsequent classification was done in a single batch to prevent exposure of individual patient data. We employed OpenAI’s latest GPT-4o model to classify these entities into relevant SDoH categories following the structure outlined in Supplementary Table 1, which aligned with the five main categories from Healthy People 2030 (https://odphp.health.gov/healthypeople/priority-areas/social-determinants-health/literature-summaries). The rationale for this classification process is that if a term does not align with any of the established SDoH categories, it is unlikely to represent a genuine social determinant of health. Therefore, in the first classification step, GPT-4o was prompted to assign entities to one of the five categories. Any entity that did not fit was treated as non-SDoH and removed from the list. In the second step, GPT-4o further classified the remaining entities into more specific subcategories, expanded from SDoH literature summaries. This dual-layer filtering ensured that non-SDoH factors were thoroughly excluded, increasing the precision of the extracted entities.

#### PubMed literature KG

First, we collected a total of 12,773 articles related to AD and SDoH from PubMed search engine (2019-2024, excluding preprints) with queries in the form of combinations of “Alzheimer’s disease” with each of the following keywords “SDoH,” “community,” “economics,” “education,” “environment,” “healthcare,” “neighborhood,” and “social.” Searches were limited to articles published within the past 5 years (2019-2024), excluding preprints. Our analysis was conducted on the abstracts of these articles. The list of PMIDs for all collected articles is provided in our GitHub repo.

As illustrated in Figure 1A, for each collected PubMed abstract, we use Pubtator 3.0,^37^ a biomedical text-mining tool, to extract six types of biomedical entities (i.e., genes, diseases, chemicals, mutations, species, and cell lines). For each pair of a biomedical entity and a SDoH entity, we assessed their relationship using two methods: GPT-4o and co-occurrence analysis. To identify the relationship, we prompted GPT-4o to read the abstract and determine the potential connection between the two entities. Additionally, to quantify their relevance, we calculated a co-occurrence score for each pair. This score is defined as the ratio of the number of sentences in which both entities appear together to the higher of the two numbers of sentences in which only one of the two entities appears. Since the score is essentially a frequency metric, we set 0.5 as a filtering threshold to retain only triplets (biomedical entity, relation, SDoH entity) that were contextually relevant to form our PubMed Literature Knowledge Graph.

#### MIMIC-III KG

For the 686 selected patients, we extracted each patient’s disease and drug usage information from the DIAGNOSES_ICD and PRESCRIPTIONS tables, respectively. Additionally, six key attributes—age, ethnicity, religion, marital status, gender, and insurance—were obtained from the ADMISSIONS and PATIENTS tables to provide supplementary data. The SDoH factors for each patient were retrieved from the discharge summaries within the NOTEEVENTS table. By connecting these various entity types for each patient and excluding triplets with a frequency of less than 10, we constructed a population-level MIMIC-III knowledge graph.

#### Knowledge graph integration

To build the final comprehensive knowledge graph, we integrated the PubMed Literature KG and the MIMIC-III KG. However, as the biomedical entities in Pubtator 3.0 and MIMIC-III use different identifiers, alignment was required before integration. We first identified the common biomedical entity types—Drug and Disease—across both datasets. For each entity of these types, we mapped it to its Concept Unique Identifier (CUI) from the Unified Medical Language System.^38^ Entities within the same type were aligned by matching their CUIs. For any nodes that could not be matched through CUIs, we retained their original identifiers to ensure that no valuable information was lost during the integration process.

### Graph-based knowledge discovery

In this section, we will present our methods to further integrate the well-established biological KG, PrimeKG, to construct our SDoHenPKG. We will also describe the performance comparison of the two KGs by evaluating their link prediction results based on graph neural networks. The workflow for this part is shown in Figure 1B.

Link prediction is widely used to infer new interactions from existing network data.^25^^,^^39^ The principle of “guilt by association”—which underpins the topological organization of networks—suggests that the structure of existing connections can reliably predict missing edges.^40^ Assuming SDoH provides novel and helpful information to biological knowledge graphs, we expect improved accuracy when predicting existing edges and more meaningful results when predicting non-existing edges. To utilize the SDoH information in our augmented KG for novel knowledge discovery, we integrated it with a widely used general biological KG, PrimeKG,^31^ following the method described earlier. We designed three link prediction experiments to compare performance on PrimeKG alone versus the combined graph with our AD-related SDoH information.

- Random Mask Task: In this task, we randomly masked a subset of edges within the graph to assess the general link prediction performance. This evaluation measured the overall robustness and accuracy of the augmented knowledge graph with SDoH data integrated.
- Targeted Genes Task: Here, we specifically masked gene-gene edges connected to a set of targeted immune genes most strongly associated with AD. The aim was to determine how well the SDoH-augmented graph can recover these crucial biological relationships, which are key to understanding AD.
- Exploratory Prediction Task: In this task, we predicted edges (connecting SDoH and gene nodes) that do not currently exist in the graph, representing potential relationships to be explored in future research. Given the extensive experimentation and time required to study gene-AD relationships through conventional biological methods, graph-based analysis offers a promising avenue to infer new interactions efficiently^41^^,^^42^. This task demonstrated the potential of the SDoH-enhanced graph to facilitate the discovery of novel insights and knowledge in the AD domain.

For the link prediction experiments, we trained a GCN^43^ for each of PrimeKG and SDoHenPKG, respectively, to learn embedded representations from both node features and graph topology. The model was trained to generate node embeddings that produce higher scores (i.e., cosine similarity here) for positive (i.e., existing) edges. Both input and output node feature dimensions were set to 50, and the model was trained for 250 epochs. To compare the link prediction results between PrimeKG and SDoHenPKG, we utilized Mean Reciprocal Rank (MRR),^44^^,^^45^ a standard evaluation metric in knowledge graph completion (i.e., link prediction) representing the average of the reciprocal ranks of a positive edge (existing edge in the KG) over negative edges (non-existing edges). The higher the MRR the better as one would want the positive edges ranked higher than the negative edges. Each experiment was conducted fifteen times per relation to ensure robustness. The experiments were performed on an Nvidia A40 GPU, using Pytorch and the Deep Graph Library (https://www.dgl.ai/) for the GCN implementation.

As mentioned earlier in the dataset section, for the Exploratory Prediction Task, to verify the findings of our KG link prediction results, we collected proteomics data and single-cell RNA-seq data to examine the top AD-related genes inferred by our exploratory analysis. For the aforementioned AMP-AD and ROSMAP proteomics data,^33^^,^^34^ we used linear regression for the differential protein abundance analysis and a threshold of 0.05 for the *p*-values (adjusted by the Benjamini-Hochberg multiple comparison corrections on the proteins), to identify proteins and corresponding genes with significantly different expression between the AD and control groups. The resultant genes were further examined in SEA-AD single-cell RNA-seq data for their gene expression. The expression levels of these genes across various cell types, including immune cells, astrocytes, endothelial cells, oligodendrocytes, and neuronal subtypes, are analyzed for control versus AD conditions to investigate their potential roles in disease pathology.

## Results

### Graph statistics

As described in the methods section and shown in Figure 1, there are five knowledge graphs involved in the study. Table 1 provides a summary of the key statistics for all the knowledge graphs used in this study. Below is a detailed breakdown of the individual graphs:

**Table 1.** Statistics of the knowledge graphs used in our study.

Graph	Literature KG	MIMIC KG	SDoH KG	PrimeKG	SDoHenPKG
**# nodes**	2,117	7,932	10,049	129,356	139,304
**# edges**	4,089	923,389	925,969	5,847,652	6,780,101

*Note*. KG = knowledge graphs; SDoH = social determinants of health.

- Literature KG: Built from PubMed literature, reflected the intersection of AD research with SDoH as identified through scientific publications.
- MIMIC KG: Constructed from the MIMIC-III dataset and incorporated patient-level attributes. Focused on SDoH of patients with AD or related diagnoses.
- SDoH KG: The integration of the Literature KG and the MIMIC KG.
- PrimeKG: A pre-existing biomedical knowledge graph. Initially, it contained 8,100,498 edges, but we removed all backward edges (i.e., edges with mirrored relationships) to align with our graph structure.
- SDoHenPKG: The fusion of PrimeKG and SDoH KG. It can be viewed as PrimeKG extended with additional SDoH knowledge and enriched patient-level information.

### Random mask task

To evaluate the general performance of link prediction, we compared the performance of PrimeKG and SDoHenPKG and aimed to determine whether the addition of SDoH knowledge improved link prediction performance. In this experiment, we focused on seven target relations in both KGs as listed in the first column of Table 2 by considering six main types of entities/nodes: Drug, Disease, Gene, Effect/Phenotype, Anatomy, and Biological Process nodes and seven types of relations/edges connecting the nodes: edges for Disease nodes to Gene nodes, Disease to Drug, Disease to Effect, Gene to Anatomy, Drug to Effect, Gene to Biological Process, and Drug to Gene. Other types of edges in the KGs are either much fewer in number or irrelevant to SDoH-AD research. For each of the seven relations, we randomly masked 20% of the edges in each KG to assess the model’s ability to predict these masked links. Table 2 shows the average MRRs and standard deviations over fifteen experiment runs per target relation, with higher MRR values indicating better prediction accuracy on the masked edges. The difference between PrimeKG and SDoHenPKG results was assessed for statistical significance. *p-*values were computed for each relation by both a two-tailed paired *t*-test and a nonparametric two-tailed Wilcoxon signed-rank test, that is, with and without the normality assumption of the data to ensure robustness, followed by a Bonferroni adjustment on the number of relations for multiple testing corrections. For all seven relations, the MRR values of SDoHenPKG demonstrated a consistent improvement compared to PrimeKG, in which 4 out of 7 were significant (both *t*-test and Wilcoxon *p* ≤ .05) after multitest correction, as shown in Table 2. This result indicates that SDoHenPKG outperformed PrimeKG not just by chance, and the integration of SDoH information indeed provided valuable context for inferring missing links. Moreover, compared to a related previous work^24^—where only literature mining was used, and no multitest correction and nonparametric test was performed—the current study presented a more solid and rigorous result.

**Table 2.** Results of the random mask task across seven relations.

Relation	MRR PrimeKG	MRR SDoHenPKG	*t*-test	Wilcoxon
	Mean ± *SD*	Mean ± *SD*	*p*	*p*
**Disease to Gene**	0.703 ± 0.012	0.723 ± 0.011	6.32e-4***	.002**
**Disease to Drug**	0.733 ± 0.017	0.746 ± 0.009	.021*	.002**
**Disease to Effect**	0.7189 ± 0.010	0.729 ± 0.008	.012*	.010 **
**Gene to Anatomy**	0.960 ± 0.001	0.961 ± 0.000	.184	.15
**Drug to Effect**	0.926 ± 0.003	0.928 ± 0.002	.059	.105
**Gene to Biological Process**	0.744 ± 0.004	0.748 ± 0.006	.157	.126
**Drug to Gene**	0.877 ± 0.010	0.886 ± 0.009	.020*	.001***

*Note*. MRR = mean reciprocal rank; *SD* = standard deviation. For each relation, the mean and *SD* of MRRs are reported; the *p-*values are computed from a paired t-test and a Wilcoxon signed-rank test, respectively, both two-tailed with Bonferroni multitest corrections. Values in the MRR SDoHenPKG column are shown in bold as they correspond to the results of our proposed KG, which consistently achieved higher performance than the baseline (PrimeKG).

* *p* ≤ .05.

** *p* ≤ .01.

*** *p* ≤ .001.

### Targeted gene mask task

This task evaluates how well the integration of SDoH data can enhance the recovery of AD-related biological relationships. We selected 23 immune genes (listed in the header of Table 3) that are highly associated with AD.^46^ For each gene, we used the functional protein-protein association networks database STRING v12.0^47^ to identify its experimentally validated connections with other genes. These validated interactions formed the masked datasets used for this task. We followed the same experimental setup as the Random Mask Task above, comparing the link prediction performance between PrimeKG and SDoHenPKG. The results, summarized in Table 3, showed that SDoHenPKG exhibited consistent advantages over PrimeKG, achieving higher MRR values across all 23 genes. After multitest corrections on the number of genes, 6 out of the 23 genes still exhibited statistically significant improvements for both the *t*-test and the Wilcoxon test, and around half (11) showed significant improvements for at least one of the tests. Notably, these include the APOE gene (*t*-test *p = *.013*, Wilcoxon *p = *.008**), which is one well-known risk factor associated with AD.

**Table 3.** Link prediction result for AD genes.

Gene	MRR PrimeKG	MRR SDoHenPKG	*t*-test	Wilcoxon
	mean ± *SD*	mean ± *SD*	*p*	*p*
**ABCA7**	0.832 ± 0.092	0.944 ± 0.079	.0034**	.021*
**ABI3**	0.627 ± 0.102	0.694 ± 0.088	.171	.126
**ADAMTS1**	0.415 ± 0.139	0.531 ± 0.201	.013*	.025*
**APOE**	0.743 ± 0.047	0.807 ± 0.042	.013*	.008 **
**BIN1**	0.757 ± 0.042	0.804 ± 0.040	.050*	.071
**C7**	0.713 ± 0.122	0.830 ± 0.067	.013*	.018*
**CASS4**	0.442 ± 0.059	0.505 ± 0.065	.016*	.077
**CD2AP**	0.717 ± 0.033	0.740 ± 0.035	.064	.105
**CD33**	0.710 ± 0.068	0.770 ± 0.063	.333	.087
**CLNK**	0.807 ± 0.127	0.911 ± 0.086	.13	.151
**CLU**	0.825 ± 0.042	0.881 ± 0.045	.041*	.018*
**CR1**	0.503 ± 0.117	0.615 ± 0.124	.117	.037*
**EPHA1**	0.738 ± 0.086	0.793 ± 0.060	.171	.058
**INPP5D**	0.762 ± 0.076	0.811 ± 0.033	.095	.029*
**MEF2C**	0.876 ± 0.044	0.908 ± 0.047	.227	.335
**PICALM**	0.760 ± 0.065	0.811 ± 0.064	.305	.211
**PLCG2**	0.884 ± 0.072	0.919 ± 0.056	.215	.297
**PTK2B**	0.894 ± 0.030	0.934 ± 0.036	.011*	.023*
**SHARPIN**	0.768 ± 0.038	0.819 ± 0.045	.021*	.058
**SORL1**	0.541 ± 0.040	0.575 ± 0.050	.083	.071
**SPI1**	0.903 ± 0.036	0.949 ± 0.038	.075	.058
**TREM2**	0.407 ± 0.078	0.494 ± 0.083	.116	.247
**TREML2**	0.560 ± 0.099	0.626 ± 0.106	.147	.105

*Note.* AD = Alzheimer’s disease; MRR **=** mean reciprocal rank; *SD* = standard deviation. For each gene, the mean and *SD* of MRRs are reported; the *p-*values are computed from a paired *t*-test and a Wilcoxon signed-rank test, respectively, both two-tailed with Bonferroni multitest corrections. Values in the MRR SDoHenPKG column are shown in bold as they correspond to the results of our proposed KG, which consistently achieved higher performance than the baseline (PrimeKG).

* *p* ≤ .05.

** *p* ≤ .01.

### Exploratory prediction task

#### Our link prediction revealed potential SDoH-genes relations previously unknown

After demonstrating the efficacy of our framework via random and targeted link predictions, we applied the framework to explore potentially novel links that are currently uncovered. In this task, we utilized the complete SDoHenPKG knowledge graph as the training set to predict the probability of potential links between unconnected gene nodes and SDoH nodes. We focused on the top 0.0001‰ predictions with the highest probabilities, and we identified 758 genes likely influenced by SDoH and related to AD. We then examined these genes with more validation steps next.

#### Proteomics analysis validated the predicted novel relations

**Figure 2.**
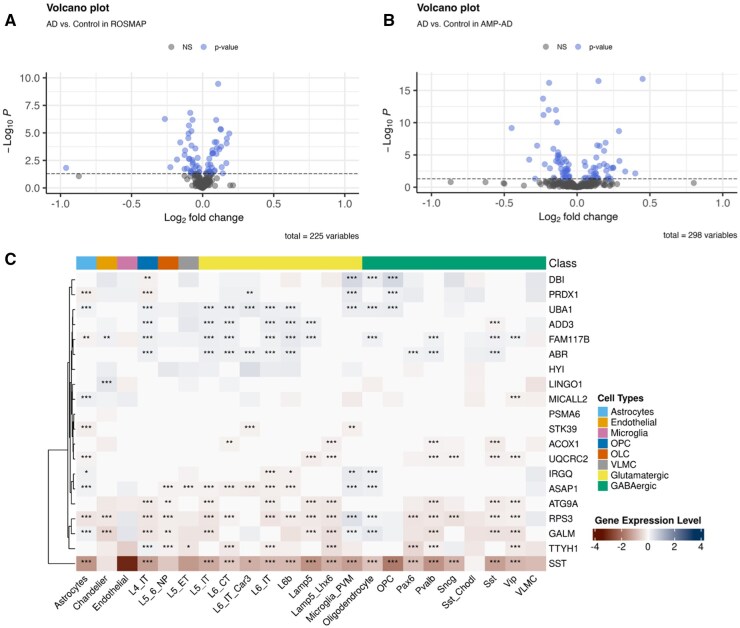
Validation of exploratory prediction results on multimodal biological data. (A and B) Differential abundance proteomics analysis volcano plot comparing AD versus Control in the AMP-AD and ROSMAP datasets, respectively, with log_2_ fold change (Log_2_FC) as x-axis and −log_10_(*p-*value) as y-axis. Proteins with significant *p*-values are in blue color, while those with non-significant *p-*values are in gray color. (C) Single-cell RNA-seq heatmap of SDoH-related gene expression across immune cells, GABAergic (i.e., inhibitory) and Glutamatergic (i.e., excitatory) neurons. The color gradient indicates expression levels (blue for upregulation and red for downregulation), with statistical significance denoted by symbols (* *p* < .05, ** *p* < .01, *** *p* < .001). There are multiple subclasses with identified markers for neuronal layers. LT: intratelencephalic, NP: near projecting, CT: corticothalamic, L4, L5, and L6 refer to layer-specific neuronal classes. Abbreviation is also used if there is a space limit. OLC: Oligodendrocyte, OPC: Oligodendrocyte precursor cells, VLMC: Ascular leptomeningeal cells. Hierarchical clustering is applied to the expression of each gene across all cell types, grouping genes that share similar expression levels. In the dendrogram (left), shorter branch lengths indicate greater similarity, so genes with highly correlated expression levels cluster together.

Here, we first validated the 758 genes for their AD relations through corresponding proteomics analysis. As introduced in the methods section, we performed differential protein abundance analysis of AD vs. control on AMP-AD and ROSMAP proteomics datasets. As a result, we identified 59 proteins with significantly different expression from 298 overlapped proteins of interest available in the AMP-AD dataset and 42 proteins with significantly different expression from 225 overlapped proteins of interest in the ROSMAP dataset. From those proteins identified in each dataset, there is a consensus of 20 proteins with differential expression in both AMP-AD and ROSMAP data. Volcano plots in Figure 2A, B illustrate the differential abundance of the proteins of interest along with their log fold changes.

#### RNA-seq analysis provided additional evidence for the top candidates from proteomics

Furthermore, for the candidate proteins identified from the proteomics analysis above, we examined their corresponding gene expressions in SEA-AD single-cell RNA-seq data. At the proteomics level, 20 genes were differentially expressed in both the ROSMAP and AMP-AD datasets. At the mRNA level, the expression levels of these genes across various cell types were analyzed for AD versus control to investigate their potential roles in disease pathology. Specifically, we focused on two main subclusters of neurons, glutamatergic and GABAergic neurons, with the functions of activation and suppression, respectively. As a result, Figure 2C shows a heatmap using the log-transformed normalized fold changes for each gene and converted values to z-scores within each cell type. We again adjusted *p*-values by the Benjamini-Hochberg multitest correction procedure to determine the significance of the expression differences. As shown in Figure 2C, SST, ATG9A, TTYH1, GALM, RPS3, and UQCRC2 genes showed pronounced downregulation across multiple neuronal subtypes, especially in inhibitory neurons, while UBA1, ASAP1, IRGQ, GALM, and RPS3 showed significant upregulation in glial populations. Additionally, ABR, ADD3, UBA1, and FAM117B were upregulated in layer-specific neurons, highlighting specific patterns of expression that correspond to layer-specific functional roles within the cortex. We will interpret the findings in greater detail in the next section.

## Discussion and implications

In this study, we developed a novel knowledge graph that integrates SDoH from both literature and EHR data to enhance link prediction in AD-related biomedical networks. To the best of our knowledge, this study presents the first-ever exploration to integrate literature and EHR-derived SDoH information together with biological knowledge for AD research. This heterogeneous data source allows for a comprehensive representation of the relationships between SDoH factors and biomedical entities, providing a richer context that aids in uncovering novel insights.

Our experimental results demonstrated that the new SDoH-enriched knowledge graph (SDoHenPKG) outperformed PrimeKG alone across various link prediction tasks. Notably, our analyses revealed improved prediction accuracy and statistical significance for AD-related relationships, particularly in tasks targeting high-priority AD genes. This improvement supported the hypothesis that SDoH factors can provide valuable contextual information in biomedical networks, enhancing the model’s ability to infer meaningful biological relationships. Furthermore, the inclusion of EHR data alongside literature sources strengthened the robustness of our graph by grounding predictions in patient-level data, which is often more reflective of real-world disease dynamics.

In the Exploratory Prediction Task, we tried to uncover potential novel SDoH-gene interactions by predicting non-existent edges in the current SDoHenPKG. From the top predictions, we identified genes likely influenced by SDoH and related to AD. SDoH may affect these genes by influencing their expressions through chronic stress, poor nutrition, and environmental exposures, thereby exacerbating gene dysfunction and contributing to increased risks in the development and progression of AD.^48^^,^^49^ Subsequent validations using proteomics and single-cell RNA-seq data were performed to find differential expression signals in the AD case and control groups. As presented in the Results section, the final results of differential expression across cell types and neuronal layers underscored the potential cell-type-specific roles of the identified SDoH-influenced genes in AD progression. An interesting finding here is the observed downregulation of SST at both RNA and protein levels. The SST gene encodes the somatostatin peptide, a neurotransmitter that regulates neurotransmission and neuroendocrine activity.^50^ Studies indicate that somatostatin levels are markedly reduced in the brains of AD patients.^51^^,^^52^ The downregulation of SST might affect Aβ clearance by deactivating neprilysin, an enzyme responsible for Aβ degradation.^53^ As an inhibitory neurotransmitter, the SST protein downregulation in inhibitory neurons may cause an excitatory-inhibitory imbalance, which may cause synaptic dysfunction and cognitive decline in AD.

Although proteomics and single-cell RNA sequencing data robustly support the gene-SDoH connections inferred from our knowledge graph analysis, these findings are correlational and incapable of determining causality between social determinants of health-related genes and AD pathology. The observed relationships may be confounded by other biological or environmental variables. In vitro functional tests, such as gene knockdown or overexpression in neuronal or glial cell models, could evaluate whether the modulation of important candidate genes like SST or UBA1 affects molecular pathways or cellular phenotypes pertinent to AD. Clinical validation, including the analysis of patient-level clinical data that correlates SDoH exposures with biomarker levels or illness development, may yield real-world evidence substantiating the biological effects of gene-SDoH interactions.

The integrated knowledge graph, SdoHenKG, which combines SDoH with biomedical knowledge related to AD, opens promising avenues for future research. First, given the nonmedical nature of SDoH, this type of KG resource can be leveraged to improve both individual and population-level health outcomes in real-world settings. In clinical practice, for example, this resource could serve as a decision-support tool by enabling early identification of individuals at risk and guiding tailored preventive interventions based on comprehensive social and biological risk profiles. At the population level, the KG’s insights into SDoH-AD relationships can inform public health planning and policy-making by guiding community interventions, resource allocation, and health equity initiatives.^54^^,^^55^ Besides, another application aspect involves managing potential knowledge conflicts or inconsistencies arising as new studies and discoveries emerge. Our SDoHenKG can serve as a dynamic repository for monitoring emerging research, since its construction and maintenance are largely automated through LLM and NLP approaches. It can be adapted to be used for detecting and solving conflicting findings, particularly in the rapidly evolving domain of AD and SDoH research. Moreover, the integration of SDoH data from literature and EHR alongside biological knowledge from PrimeKG is complex and may introduce challenges related to data variability and quality. Future work could explore improved methods to enhance data harmonization across heterogeneous datasets and alternative sources (e.g., the largest AD KG mentioned earlier, AlzKB,^30^ has yet to incorporate SDoH information). Additionally, while our model performed well on the current dataset supported by biological evidence, further validation on other independent datasets would be necessary to confirm the generalizability of the findings. It is also important to recognize that missing or underrepresented patient demographics, such as age, gender, and ethnicity, can impact model predictions. Incomplete demographic information or imbalances in representation may skew the analysis of SDoH and their relationship with AD. For example, underrepresentation of certain groups can lead to biased conclusions. To mitigate this, we can apply imputation techniques for missing data and ensure validation on diverse datasets to improve the model’s generalizability and fairness.^56^ Furthermore, for the backbone LLM, we also explored using a previous biomedical domain-specific language model, BioBERT,^57^ as an alternative to GPT within our framework, as illustrated in Figure 1A. However, our empirical experiments (results not shown here) revealed that BioBERT’s performance in filtering SDoH entities was severely limited, with very few entities effectively filtered (average filter rate around 4% compared with GPT-4o 70%). Given that BioBERT was developed in 2019 based on the BERT encoder architecture, this observation likely indicates that our framework relies on the advanced language-modeling capabilities of the backbone LLMs, which are crucial for accurately extracting and processing SDoH information. In future work, additional comparisons with more recent and powerful domain-specific LLMs would be highly valuable, potentially enabling further enhancements in our framework.

In conclusion, our study demonstrates that integrating SDoH with biomedical data not only improves predictive performance but also reveals new insights into the interplay between social determinants and biological pathways in AD. The proposed framework underlines the utility of combining SDoH with omics analyses, especially in complex diseases where social and biological factors are intertwined. This research sets the stage for further exploration of SDoH in biomedical networks, with potential implications for more personalized and socially aware AD interventions.

## Supplementary Material

igaf102_Supplementary_Data

## Data Availability

The EHR data used in this study are collected from the MIMIC-III Clinical Database https://physionet.org/content/mimiciii/1.4/ and the literature data are obtained from the PubMed search engine. The proteomics data we used are collected from the Accelerating Medicines Partnership for Alzheimer’s Disease (AMP-AD) Diverse Cohorts and the Religious Orders Study and Memory and Aging Project (ROSMAP), both available in Synapse (AMP-AD: https://www.synapse.org/Synapse:syn59611693; ROSMAP: https://www.synapse.org/Synapse:syn21261728). The single-cell RNA sequencing data are publicly available from the Seattle Alzheimer’s Disease Brain Cell Atlas (SEA-AD) and downloaded from https://cellxgene.cziscience.com/collections/1ca90a2d-2943-483d-b678-b809bf464c30.
