# Supplementary material for: A novel computational analysis integrating social determinants information from EHR and literature with Alzheimer’s disease biological knowledge through large language models and knowledge graphs
Source: Innov Aging. 2025 Sep 23;9(Suppl 1):S2–S13. doi: 10.1093/geroni/igaf102 (PMC12742847; doi:10.1093/geroni/igaf102)
Supplement: igaf102_Supplementary_Data [file igaf102_supplementary_data.zip › innage suppl Shang et al (supplement).docx]

***Innovation in Aging* Supplementary Material: Shang et al.** **A novel computational analysis integrating social determinants information from EHR and literature with Alzheimer’s disease biological knowledge through large language models and knowledge graphs.**

**Supplementary Table 1**: SDoH type and subtypes.

| Health Care Access and Quality | Education Access and Quality | Social and Community Context | Economic Stability | Neighborhood and Built Environment |
| --- | --- | --- | --- | --- |
| Access to Health Services | Early Childhood Development and Education | Civic Participation | Employment | Access to Foods That Support Healthy Dietary Patterns |
| Access to Primary Care | Enrollment in Primary Education | Racism and Discrimination | Income | Crime and Violence |
| Health Literacy | Enrollment in Secondary Education | Incarceration | Wealth | Environmental Conditions |
| Health Insurance | Enrollment in Higher Education | Social Cohesion | Poverty | Quality of Housing |
|  | Language and Literacy | Involved in illegal activities | Transportation | Homelessness |
|  | Uneducated |  |  | Food Insecurity |
|  |  |  |  | Quality of Neighborhoods |

**Supplementary Table 2**: Demographic details of the AMP-AD and ROSMAP subjects we used in this study.

| **Variable** | **AMP-AD** | | **ROSMAP** | |
| --- | --- | --- | --- | --- |
|  | **AD** | **Control** | **AD** | **Control** |
| ***N*** | 645 | 249 | 108 | 84 |
| **Age: mean (SD)** | 81.4 (9.2) | 75.5 (14.4) | 88.2 (3.1) | 85.2 (5.1) |
| **Female Sex: N (%)** | 386 (59.8) | 140 (56.2) | 81 (75.0) | 50 (59.5) |
